# Universal Genetic Testing vs. Guideline-Directed Testing for Hereditary Cancer Syndromes Among Traditionally Underrepresented Patients in a Community Oncology Program

**DOI:** 10.7759/cureus.37428

**Published:** 2023-04-11

**Authors:** Jeremy C Jones, Michael A Golafshar, Tucker W Coston, Rohit Rao, Ewa Wysokinska, Elizabeth Johnson, Edward D Esplin, Robert L Nussbaum, Brandie Heald, Margaret Klint, Kathleen Barrus, Pedro L Uson Jr., Cuong C Nguyen, Gerald Colon-Otero, Tanios S Bekaii-Saab, Roxana Dronca, Katie L Kunze, N. Jewel Samadder

**Affiliations:** 1 Hematology & Oncology, Mayo Clinic, Jacksonville, USA; 2 Health Policy, Mayo Clinic, Phoenix, USA; 3 Medical Affairs, Invitae Corporation, San Francisco, USA; 4 Clinical Genomics, Mayo Clinic, Phoenix, USA; 5 Hematology & Oncology, Mayo Clinic, Phoenix, USA; 6 Gastroenterology and Hepatology, Mayo Clinic, Phoenix, USA

**Keywords:** screening strategy, hereditary cancer syndromes, homologous recombination deficiency, lynch syndrome, germline testing

## Abstract

Background

Detection of pathogenic germline variants (PGVs) has implications for cancer screening, prognosis, treatment selection, clinical trial enrollment, and family testing. Published guidelines provide indications for PGV testing, determined by clinical and demographic factors, but their applicability in an ethnically and racially diverse community hospital population is unknown. This study describes the diagnostic and incremental yield of universal multi-gene panel testing in a diverse population in a community cancer practice.

Methods

We completed a prospective study of proactive germline genetic sequencing among patients with solid tumor malignancies at a community-based oncology practice in downtown Jacksonville, FL, between June 2020 and September 2021. The patients were unselected for cancer type, stage, family history, race/ethnicity, and age. PGVs identified using an 84-gene next-generation sequencing (NGS) tumor genomic testing platform were stratified by penetrance. National Comprehensive Cancer Networks (NCCN) guidelines determined incremental PGV rates.

Results

Two hundred twenty-three patients were enrolled, with a median age of 63 years, 78.5% female. 32.7% were Black/African American, and 5.4% were Hispanic. 39.9% of patients were commercially insured, Medicare/Medicaid insured 52.5%, and 2.7% were uninsured. The most common cancers in this cohort were breast (61.9%), lung (10.3%), and colorectal (7.2%). Twenty-three patients (10.3%) carried one or more PGVs, and 50.2% carried a variant of uncertain significance (VUS). Though there was no significant difference in the rate of PGVs based on race/ethnicity, African Americans were numerically more likely to have a VUS reported than whites (P=0.059). Eighteen (8.1%) patients had incremental clinically actionable findings that practice guidelines would not have detected, which was higher in non-whites.

Conclusions

In this racially/ethnically and socioeconomically diverse cohort, universal multi-gene panel testing (MGPT) increased diagnostic yield over targeted guideline-informed testing. Rates of VUS and incremental PGV were higher in non-white populations.

## Introduction

Inherited genetic factors play a significant role in the risk of developing multiple cancers. [1,2] Detection of pathogenic or likely-pathogenic germline variants (PGVs) has implications for cancer screening, prognostication, therapy selection, clinical trial eligibility, and family testing recommendations. Prior studies have estimated the prevalence of germline cancer susceptibility in patients with various solid tumors. However, these studies have mainly been limited to white European populations recruited at academic medical centers, registry populations, and genetic testing companies, likely leading to bias.

Selection for genetic testing has traditionally been based on clinical, tumor, and family history factors stipulated in practice guidelines. Current guidelines recommend testing for high-risk individuals based on personal or family history and various disease-associated risk factors such as age at diagnosis and certain pathologic features. The research underlying current guideline criteria is based mainly on studies enriched for individuals of Northern European descent, with a relative paucity of data from populations historically underrepresented in clinical research. Accordingly, the sensitivity of these criteria has yet to be tested in racially and ethnically diverse populations. 

Our group has previously described experience with universal genetic testing versus guideline-directed testing in unselected patients with cancer at a large multi-site cancer center. [3] In this work, we found that nearly 50% of patients who were ultimately found to have PGVs did not meet existing guidelines for testing, and a significant portion had a change of management based on their genetic testing results. Despite these findings, questions regarding the feasibility of such an approach outside an academic medical institution and with a more diverse patient population still need to be answered.

We designed a prospective cohort study of hereditary cancer testing with a multi-gene panel in an urban community-based, embedded cancer center with a greater proportion of patients from traditionally under-represented racial and ethnic backgrounds. We report the diagnostic yield of testing and the incremental yield found with a broad universal testing strategy compared with a guideline-based targeted strategy [4]. 

This study was previously presented as a poster at the 2022 ASCO Annual Meeting on June 2, 2022.

## Materials and methods

We completed a prospective study of proactive germline genetic sequencing among patients with solid tumor malignancies at a community-based oncology practice in downtown Jacksonville, FL, between June 29, 2020, and September 15, 2021 (Inherit trial). This study was approved by the Mayo Clinic Institutional Review Board (IRB 19-011472). All patients provided written informed consent. Data were deidentified except to study investigators. The study followed the Strengthening the Reporting of Observational Studies in Epidemiology (STROBE) reporting guidelines.

Adult patients with new or active solid-tumor malignancies receiving medical oncology care at the Mayo Clinic embedded community oncology clinic at St. Vincent’s Riverside Hospital were enrolled during the study period. Patients with hematologic malignancies and undergoing active surveillance with no evidence of active malignancy were excluded. Patients were identified from daily central scheduling lists by research coordinators. Patients were unselected for age, race/ethnicity, family history of cancer, cancer type, or stage of the disease. Germline sequencing using a next-generation sequencing panel of 84 genes on the Invitae Multi-Cancer panel was free. A certified genetic counselor reviewed all test results and results disclosed to the patient. Patients with detected PGVs were also contacted and invited for formal genetic counseling. Clinical, demographic, family history and pathologic information were collected on all patients from a personal interview or electronic medical records. 

Sequencing, variant calling, and result reporting

Whole gene sequencing, deletion, and duplication analysis of all coding exons, +/- 20 base pairs of flanking intron, and other special targets, and variant interpretations were performed at Invitae as previously described [5]. PGVs were classified as high (relative risk > 4), intermediate (relatively risk 2-4), or low (relative risk < 2) penetrance or recessive medically actionable variants [6].

Comparison to guideline-based testing

2020 National Comprehensive Cancer Network (NCCN) guidelines were used to determine whether genetic testing was indicated based on clinical presentation [2,7]. A PGV was considered incremental if it was detected based on the universal testing performed in this study and a) was a gene outside of those recommended on a guideline-based panel, or b) would not have been identified based on genetic testing/referral criteria on the 2020 NCCN guidelines. 

Family variant testing

Cascade family variant testing was offered at no charge to all blood relatives of affected patients with detected PGVs within 90 days of the patients’ finalized test report. Patients were informed during post-test genetic counseling of this service offered by Invitae and assisted in communicating this information to their relatives. This was via a standardized template letter and an online video describing the risk and benefits of germline sequencing.

Statistical analysis

Demographic and clinical characteristics of the cohort are presented using descriptive statistics. The prevalence of PGV and variants of unknown significance (VUS) are reported in the cohort. Where appropriate, categorical variables were compared using Pearson’s Chi-square (X2) test or Kruskal-Wallis non-parametric tests. Rates of incremental findings were compared between subgroups by X2, and proportions of germline findings were compared by stage of disease using the X2 test. All statistical tests were two-sided, and P values of less than 0.05 were considered statistically significant. Univariate logistic regression models were used to predict pathogenic germline mutation.

## Results

Cohort characteristics

Two hundred twenty-three patients were enrolled in the study, all of whom underwent genetic testing with results available for review. The distribution of age, sex, stage, cancer type, and comorbidities in these patients is shown in Tables 1, 2. The median age was 63.0 years at diagnosis (range 27 to 83 years), and 78.5% were female, which mirrored the demographics of the community practice. Breast cancer was the most common cancer diagnosis accounting for 61.9% of all patients, followed by lung cancer at 10.3%, colorectal at 7.2%, and melanoma at 4.5%. The distribution of cancer stages was as follows: Stage 0 (ductal carcinoma in situ) accounted for 9.9%, stage I 31.8%, stage II 26.9%, stage III 15.7%, and stage IV 15.7%. Race and ethnicity distributions for non-white patients included 32.7% Black or African American, 5.4% Hispanic/Latino, 3.1% Asian, and 2.6% other/chose not to disclose race. Sixty percent of patients had government insurance or were uninsured at enrollment (Medicare 43.5%, Medicaid 9.0%, Tricare/Veterans Affairs Benefits 4.9%, and uninsured 2.7%). The prevalence of body mass index (BMI) >30, diabetes, and hypertension were 45.7%, 25.1%, and 54.7%, respectively. A family history of cancer (any relative) was reported in 58.6% of participants.

**Table 1:** Clinical and Demographic characteristics of included patients 1. Pearson’s Chi-squared test, 2. Kruskal-Wallis rank sum test. Legend: VUS, a variant of uncertain significance; SD, standard deviation; BMI, body mass index

	Total	White	Black/African American	Hispanic/Latino	Other	p-value
N	223	125	73	12	13	
Germline Result						0.427^1^
Positive	23 (10.3%)	13 (10.4%)	7 (9.6%)	1 (8.3%)	2 (15.4%)	
VUS	112 (50.2%)	58 (46.4%)	44 (60.3%)	6 (50.0%)	4 (30.8%)	
Negative	88 (39.5%)	54 (43.2%)	22 (30.1%)	5 (41.7%)	7 (53.8%)	
Sex						0.065^1^
Male	48 (21.5%)	35 (28.0%)	9 (12.3%)	2 (16.7%)	2 (15.4%)	
Female	175 (78.5%)	90 (72.0%)	64 (87.7%)	10 (83.3%)	11 (84.6%)	
Age						0.076^2^
Mean (SD)	61.8 (11.4)	62.7 (11.1)	61.4 (10.8)	52.5 (14.8)	64.1 (11.5)	
Median	63.0	63.0	61.0	53.5	68.0	
Range	27.0 - 83.0	27.0 - 82.0	34.0 - 83.0	28.0 - 72.0	31.0 - 74.0	
Race / Ethnicity						<0.001^1^
White (Non-Hispanic)	125 (56.1%)	125 (100.0%)	0 (0.0%)	0 (0.0%)	0 (0.0%)	
Black or African American	73 (32.7%)	0 (0.0%)	73 (100.0%)	0 (0.0%)	0 (0.0%)	
Hispanic/Latino	12 (5.4%)	0 (0.0%)	0 (0.0%)	12 (100.0%)	0 (0.0%)	
Asian	7 (3.1%)	0 (0.0%)	0 (0.0%)	0 (0.0%)	7 (53.8%)	
Other	1 (0.4%)	0 (0.0%)	0 (0.0%)	0 (0.0%)	1 (7.7%)	
Choose Not to Disclose	5 (2.2%)	0 (0.0%)	0 (0.0%)	0 (0.0%)	5 (38.5%)	
Obesity (BMI>30)						0.049^1^
Yes	102 (45.7%)	51 (40.8%)	41 (56.2%)	7 (58.3%)	3 (23.1%)	
No	121 (54.3%)	74 (59.2%)	32 (43.8%)	5 (41.7%)	10 (76.9%)	
Hypertension						0.002^1^
Yes	122 (54.7%)	58 (46.4%)	53 (72.6%)	6 (50.0%)	5 (38.5%)	
No	101 (45.3%)	67 (53.6%)	20 (27.4%)	6 (50.0%)	8 (61.5%)	
Diabetes Mellitus						0.083^1^
Yes	56 (25.1%)	25 (20.0%)	26 (35.6%)	3 (25.0%)	2 (15.4%)	
No	167 (74.9%)	100 (80.0%)	47 (64.4%)	9 (75.0%)	11 (84.6%)	
Insurance Status						0.347^1^
Commercial	89 (39.9%)	50 (40.0%)	31 (42.5%)	5 (41.7%)	3 (23.1%)	
Medicaid	20 (9.0%)	8 (6.4%)	7 (9.6%)	2 (16.7%)	3 (23.1%)	
Medicare	97 (43.5%)	59 (47.2%)	28 (38.4%)	3 (25.0%)	7 (53.8%)	
Military/TriCare	11 (4.9%)	6 (4.8%)	3 (4.1%)	2 (16.7%)	0 (0.0%)	
Uninsured	6 (2.7%)	2 (1.6%)	4 (5.5%)	0 (0.0%)	0 (0.0%)	
Primary Cancer						0.148^1^
Breast	138 (61.9%)	70 (56.0%)	53 (72.6%)	8 (66.7%)	7 (53.8%)	
Lung	23 (10.3%)	10 (8.0%)	10 (13.7%)	0 (0.0%)	3 (23.1%)	
Colorectal	16 (7.2%)	11 (8.8%)	4 (5.5%)	1 (8.3%)	0 (0.0%)	
Melanoma	10 (4.5%)	9 (7.2%)	0 (0.0%)	0 (0.0%)	1 (7.7%)	
Pancreas	5 (2.2%)	2 (1.6%)	1 (1.4%)	1 (8.3%)	1 (7.7%)	
Prostate	5 (2.2%)	2 (1.6%)	3 (4.1%)	0 (0.0%)	0 (0.0%)	
Renal	5 (2.2%)	5 (4.0%)	0 (0.0%)	0 (0.0%)	0 (0.0%)	
Esophageal	3 (1.3%)	3 (2.4%)	0 (0.0%)	0 (0.0%)	0 (0.0%)	
Gastric	4 (1.8%)	2 (1.6%)	1 (1.4%)	0 (0.0%)	1 (7.7%)	
Ovarian	3 (1.3%)	3 (2.4%)	0 (0.0%)	0 (0.0%)	0 (0.0%)	
Appendix	2 (0.9%)	2 (1.6%)	0 (0.0%)	0 (0.0%)	0 (0.0%)	
Head/neck	2 (0.9%)	0 (0.0%)	1 (1.4%)	1 (8.3%)	0 (0.0%)	
Hepatocellular	2 (0.9%)	1 (0.8%)	0 (0.0%)	1 (8.3%)	0 (0.0%)	
Small bowel	2 (0.9%)	2 (1.6%)	0 (0.0%)	0 (0.0%)	0 (0.0%)	
Thyroid	1 (0.4%)	1 (0.8%)	0 (0.0%)	0 (0.0%)	0 (0.0%)	
Other	2 (0.9%)	2 (1.6%)	0 (0.0%)	0 (0.0%)	0 (0.0%)	
Cancer Stage at Diagnosis						0.895^1^
0/1	93 (41.7%)	49 (39.2%)	32 (43.8%)	7 (58.3%)	5 (38.5%)	
2	60 (26.9%)	34 (27.2%)	21 (28.8%)	2 (16.7%)	3 (23.1%)	
3	35 (15.7%)	22 (17.6%)	9 (12.3%)	2 (16.7%)	2 (15.4%)	
4	35 (15.7%)	20 (16.0%)	11 (15.1%)	1 (8.3%)	3 (23.1%)	
Family History of Cancer						0.039^1^
Yes	130 (58.6%)	73 (58.4%)	48 (66.7%)	3 (25.0%)	6 (46.2%)	
No	92 (41.4%)	52 (41.6%)	24 (33.3%)	9 (75.0%)	7 (53.8%)	
Missing	1	0	1	0	0	

**Table 2:** Clinical and demographic characteristics based on germline testing result 1. Pearson’s Chi-squared test, 2. Kruskal-Wallis rank sum test Legend: VUS, a variant of uncertain significance; SD, standard deviation; BMI, body mass index

	Overall	Positive	VUS	Negative	p value
N	223	23	112	88	
Sex					0.950^1^
Male	48 (21.5%)	5 (21.7%)	25 (22.3%)	18 (20.5%)	
Female	175 (78.5%)	18 (78.3%)	87 (77.7%)	70 (79.5%)	
Age					0.642^2^
Mean (SD)	61.8 (11.4)	62.0 (13.2)	62.4 (11.3)	61.1 (11.1)	
Median	63.0	67.0	63.0	61.0	
Range	27.0 - 83.0	27.0 - 77.0	31.0 - 83.0	28.0 - 82.0	
Race / Ethnicity					0.282^1^
White (Non-Hispanic)	125 (56.1%)	13 (56.5%)	58 (51.8%)	54 (61.4%)	
Black or African American	73 (32.7%)	7 (30.4%)	44 (39.3%)	22 (25.0%)	
Hispanic/Latino	12 (5.4%)	1 (4.3%)	6 (5.4%)	5 (5.7%)	
Asian	7 (3.1%)	0 (0.0%)	3 (2.7%)	4 (4.5%)	
Other	1 (0.4%)	0 (0.0%)	0 (0.0%)	1 (1.1%)	
Choose Not to Disclose	5 (2.2%)	2 (8.7%)	1 (0.9%)	2 (2.3%)	
Obesity (BMI>30)					0.014^1^
Yes	102 (45.7%)	4 (17.4%)	53 (47.3%)	45 (51.1%)	
No	121 (54.3%)	19 (82.6%)	59 (52.7%)	43 (48.9%)	
Hypertension					0.014^1^
Yes	122 (54.7%)	6 (26.1%)	64 (57.1%)	52 (59.1%)	
No	101 (45.3%)	17 (73.9%)	48 (42.9%)	36 (40.9%)	
Diabetes Mellitus					0.827^1^
Yes	56 (25.1%)	5 (21.7%)	30 (26.8%)	21 (23.9%)	
No	167 (74.9%)	18 (78.3%)	82 (73.2%)	67 (76.1%)	
Insurance Status					0.174^1^
Commercial	89 (39.9%)	5 (21.7%)	53 (47.3%)	31 (35.2%)	
Medicaid	20 (9.0%)	2 (8.7%)	7 (6.2%)	11 (12.5%)	
Medicare	97 (43.5%)	14 (60.9%)	47 (42.0%)	36 (40.9%)	
Military/TriCare	11 (4.9%)	2 (8.7%)	2 (1.8%)	7 (7.9%)	
Uninsured	6 (2.7%)	0 (0.0%)	3 (2.7%)	3 (3.4%)	
Primary Cancer					0.383^1^
Breast	138 (61.9%)	14 (60.9%)	69 (61.6%)	55 (62.5%)	
Lung	23 (10.3%)	5 (21.7%)	11 (9.8%)	7 (8.0%)	
Colorectal	16 (7.2%)	1 (4.3%)	10 (8.9%)	5 (5.7%)	
Melanoma	10 (4.5%)	0 (0.0%)	3 (2.7%)	7 (8.0%)	
Pancreas	5 (2.2%)	1 (4.3%)	2 (1.8%)	2 (2.3%)	
Prostate	5 (2.2%)	0 (0.0%)	2 (1.8%)	3 (3.4%)	
Renal	5 (2.2%)	0 (0.0%)	3 (2.7%)	2 (2.3%)	
Esophageal	3 (1.3%)	0 (0.0%)	1 (0.9%)	2 (2.3%)	
Gastric	4 (1.8%)	1 (4.3%)	3 (2.7%)	0 (0.0%)	
Ovarian	3 (1.3%)	0 (0.0%)	2 (1.8%)	1 (1.1%)	
Appendix	2 (0.9%)	0 (0.0%)	2 (1.8%)	0 (0.0%)	
Head/neck	2 (0.9%)	0 (0.0%)	1 (0.9%)	1 (1.1%)	
Hepatocellular	2 (0.9%)	0 (0.0%)	2 (1.8%)	0 (0.0%)	
Small bowel	2 (0.9%)	0 (0.0%)	0 (0.0%)	2 (2.3%)	
Thyroid	1 (0.4%)	1 (4.3%)	0 (0.0%)	0 (0.0%)	
Other	2 (0.9%)	0 (0.0%)	1 (0.9%)	1 (1.1%)	
Cancer Stage at Diagnosis					0.101^1^
0/1	93 (41.7%)	7 (30.4%)	54 (48.2%)	32 (36.3%)	
2	60 (26.9%)	10 (43.5%)	28 (25.0%)	22 (25.0%)	
3	35 (15.7%)	5 (21.7%)	11 (9.8%)	19 (21.6%)	
4	35 (15.7%)	1 (4.3%)	19 (17.0%)	15 (17.0%)	
Family History of Cancer					0.127^1^
Yes	130 (58.6%)	18 (78.3%)	63 (56.8%)	49 (55.7%)	
No	92 (41.4%)	5 (21.7%)	48 (43.2%)	39 (44.3%)	
Missing	1	0	1	0	

Variant detection

Among 223 patients, 23 patients (10.3%) harbored 27 PGVs, of whom 4 patients had more than 1 PGV detected. The most common PGVs detected were in BRCA1/2 (OMIM113705 and 600185), CHEK2 (OMIM 604373), TP53, and RAD51C, each of which occurred in 3 patients (Table 3). The PGVs could be stratified into those with high (n=12), moderate (=13), or low (n= 1) penetrance, and 1 was a carrier of a variant associated with a recessive syndrome. The rate of PGVs in patients of various self-reported races/ethnicity was Black or African American (9.6%), non-Hispanic white (10.4%), and Hispanic/Latino (8.3%) and was not statistically different. The distribution of sex, age, race/ethnicity, cancer type, and stage stratified by variant type is given in Table 2. The rate of VUS was numerically higher in Black/African Americans than in white patients, but given the relatively small sample size, this did not meet statistical significance (60.3% vs. 46.4%, P 0.059).

**Table 3:** Distribution of PGV cases by penetrance status Legend: PGV, pathogenic germline variant

Penetrance Level	PGV	Total (n = 27)
High Penetrance	BRCA1	2 (7.4%)
	BRCA2	3 (11.1%)
	CDKN2A	1 (3.7%)
	MEN1	1 (3.7%)
	PALB2	1 (3.7%)
	SDHA	1 (3.7%)
	TP53	3 (11.1%)
Moderate Penetrance	ATM	1 (3.7%)
	BRIP1	2 (7.4%)
	CDKN1B	1 (3.7%)
	CHEK2	3 (11.1%)
	HOXB13	1 (3.7%)
	MITF	2 (7.4%)
	RAD51C	3 (11.1%)
Low Penetrance	MUTYH	1 (3.7%)
Recessive Alleles	RECQL4	1 (3.7%)

Clinical implications of PGVs

Without exception, the genes in which high-penetrance PGVs were identified have published management recommendations. These recommendations include options for precision therapy or enrollment in clinical trials. Of the 23 patients with a PGV, 14 (60%) had PGVs in genes conferring potential precision therapy or clinical treatment trial eligibility (eTable 1 in the Supplement). Overall, 21/23 patients (91%) had PGVs (including low, moderate, and high penetrance genes) with potential clinical actionability, including eligibility for precision therapies, clinical trials, or published clinical management recommendations indicated by the detected PGVs.

Cascade FVT

We offered no-charge cascade family variant testing (FVT) to all blood relatives of affected participants. Within a 3-month window of their test result, there was no uptake of FVT.

Clinical genetic referral criteria

Eighteen (78%) cases had 23 incremental clinically actionable pathogenic germline findings that would not have been detected by phenotype or family-history-based testing criteria using 2020 NCCN guidelines or were outside those recommended on a panel for their primary cancer (Table 4). Of the 23 incremental PGVs, 7, 13, and 2 were categorized as high-, moderate-, or low/recessive-penetrance variants, respectively. When stratified by race, patients identified as Black/African American were likelier to have an incremental mutation than whites (85.7% vs. 76.9% incremental). However, given the small sample sizes, this difference did not meet statistical significance.

**Table 4:** Findings not predicted by 2020 NCCN Clinical guidelines: Incremental findings Legend: PGV, pathogenic germline variant; NCCN, National Cancer Care Network

	All PGV (N=23)	White (N=13)	Black/African American (N=7)	Other (N=3)
Incremental finding				
Yes	18 (78.3%)	10 (76.9%)	6 (85.7%)	2 (66.7%)
No	5 (21.7%)	3 (23.1%)	1 (14.3%)	1 (33.3%)
Did they meet NCCN testing guidelines for their primary cancer?				
Yes	10 (43.5%)	6 (46.2%)	3 (42.9%)	1 (33.3%)
No	13 (56.5%)	7 (53.8%)	4 (57.1%)	2 (66.7%)
Did they meet guidelines based on family history regardless of personal history?				
Yes	6 (60.0%)	4 (66.7%)	2 (66.7%)	0 (0.0%)
No	4 (40.0%)	2 (33.3%)	1 (33.3%)	1 (100.0%)
Did they meet guidelines based on a tumor test result?				
Yes	2 (20.0%)	1 (16.7%)	1 (33.3%)	0 (0.0%)
No	8 (80.0%)	5 (83.3%)	2 (66.7%)	1 (100.0%)
Was the PGV identified outside of the primary genes recommended for their primary cancer?				
Yes	15 (65.2%)	9 (69.2%)	5 (71.4%)	1 (33.3%)
No	8 (34.8%)	4 (30.8%)	2 (28.6%)	2 (66.7%)

Predictors of variants

In logistic regression analysis, early age of cancer diagnosis, sex, stage, and race was not associated with an increased likelihood of PGV presence (Table 5). Family cancer history in a first-degree relative was associated with an increased likelihood of having a PGV (OR 2.80, 95% CI: 1.07-8.74). The Black/African American race appeared to be associated with an increased risk of having a VUS, although the increase did not quite meet the 95% confidence limit (OR 1.753, 95% CI 0.979-3.170).

**Table 5:** Logistic regression OR and 95% CIs of patient predictors of pathogenic/likely pathogenic mutation Legend: OR, odds ratio; CI, confidence interval; ref, reference value; y, years

	OR	95% CI	p-value
Age group, y			
>=50	1.0 (ref)		
<50	1.100	0.246-3.523	0.885
Sex			
Female	1.0 (ref)		
Male	1.014	0.320-2.712	0.979
Cancer stage at diagnosis			
Early (stage 0-3)	1.0 (ref)		
Advanced (stage 4)	0.222	0.012-1.114	0.148
Family History of cancer in a first-degree relative			
No	1.0 (ref)		
Yes	2.796	1.068-8.736	0.050
Race			
White	1.0 (ref)		
Black/African American	0.914	0.329-2.348	0.855

## Discussion

This prospective study of universal germline sequencing in a cohort of traditionally under-represented patients with cancer at a community-based cancer center presents data to inform the practice of genetic testing. The prevalence of PGVs was no different between different patient populations based on race or ethnicity. In contrast, the VUS rate and guideline-based testing performance were strikingly different in minority populations.

The overall PGV prevalence in this patient population was 10.4%, consistent with prior studies, including our INTERCEPT study. A multi-clinic study from Northern California that enrolled 41% Hispanic population similarly identified a PGV in 12% using a 25-28 gene hereditary cancer panel [8]. There are a few other studies that have prospectively enrolled a large number of unselected minority patients into genetic testing/evaluation studies [9,10]. In a retrospective study of nearly 200 African Americans with prostate cancer, 10% had a PGV which was not statistically different from the Caucasian/white population [11].

A striking result was that over three-quarters of the PGVs identified would remain undetected using the 2020 NCCN clinical practice guidelines. This “incremental PGV” rate was even higher in African American/Black population, suggesting that the current guidelines are likely based on historical data derived from European and North American populations with less diversity. These data raise the possibility that current guidelines unintentionally perpetuate and potentially aggravate healthcare disparities. This finding highlights the limitations of clinical and guideline-based risk assessment consistent with prior studies while also expanding our understanding of their performance in under-represented populations.

**Figure 1:**
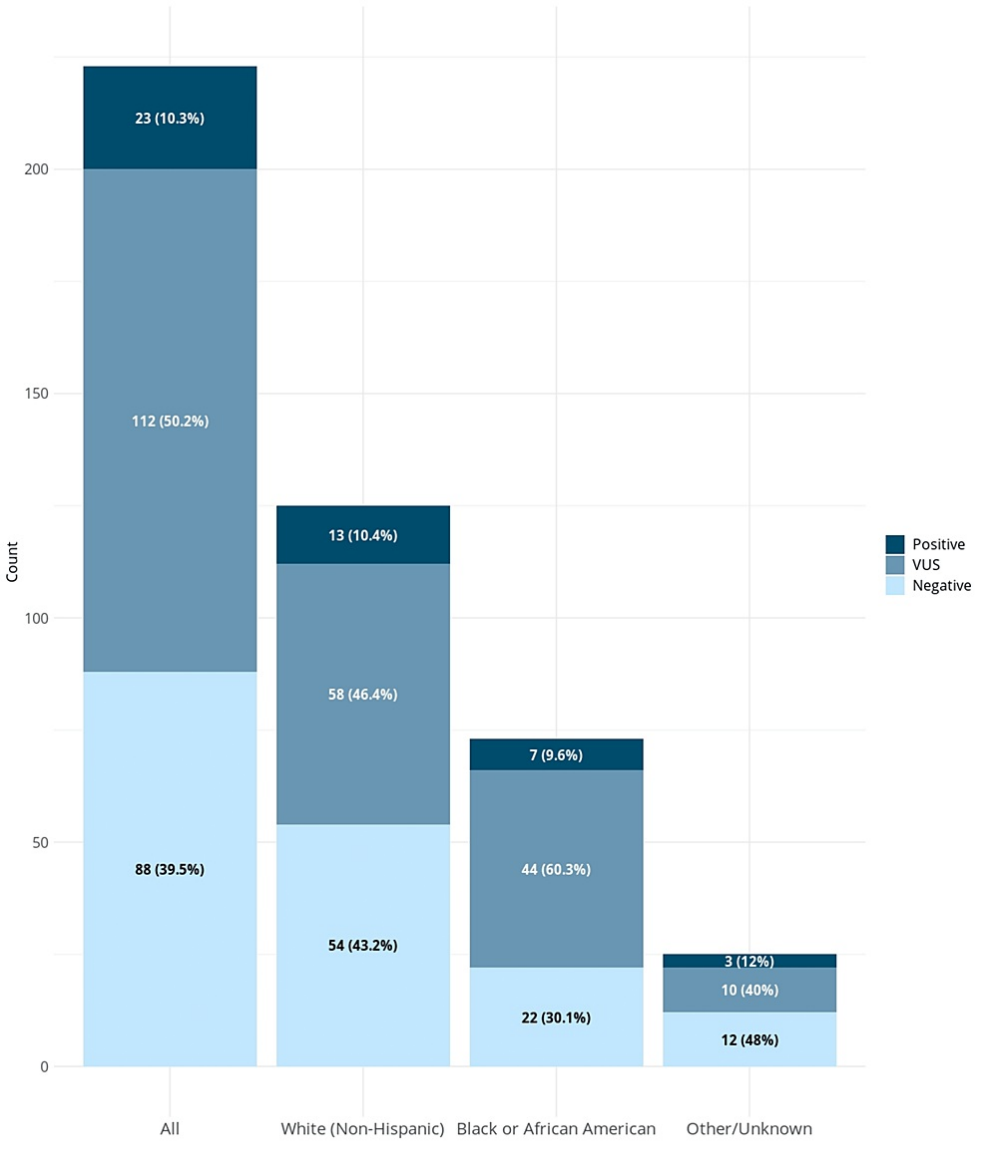
Distribution of test results based on reported race Legend: VUS, variant of uncertain significance

There are limited data on genetic testing findings among non-White populations undergoing multi-gene panel testing (MGPT). In our diverse cohort, the rate of VUS in African Americans/Blacks was much higher than in Whites (62% vs. 46%). This may reflect the historical emphasis of genetic research on White European populations, resulting in lag in the progress of variant interpretation in minority populations (demonstrated in Figure 1) [12,13]. This lack of diversity has wide-reaching effects on all patients but is particularly troublesome for minority populations with cancer. For patients from racial and ethnically diverse populations, fewer sequencing data points result in less knowledge of normal and pathologic variants. These patients are more likely to have either indeterminate (VUS) results for rare variants that may or may not have pathogenic potential, leading to less conclusive genetic sequencing results [14]. This finding underscores the importance of investigating the use and implications of MPGT in traditionally under-represented populations. 

Concerns have been raised about the high rates of VUSs identified when employing MGPT. Our VUS rate of 45% is consistent with other studies that have employed moderately to large MGPT. Including genes with unknown or unclear clinical relevance in some of these panels may also lead to invasive procedures or morbid prophylactic operations with unclear benefits. Though these are potential limitations of MGPT, decreasing testing costs has allowed the broader application of comprehensive panels that can identify clinically relevant PGVs, many with precision therapeutic implications, that may otherwise be missed due to limited family history or disparities in insurance coverage.

Cascade family variant testing (FVT) has been the backbone of cost-effectiveness analysis, supporting the benefit of universal genetic testing in several cancer types. Several studies have shown sub-optimal uptake of FVT. In the current study, even with the offer of no-charge FVT, there was no uptake by family members noted up to 90 days after completion of the study. Multiple barriers leading to low uptake of FVT may be at play, including limited understanding by at-risk relatives, the technical nature of reports from genetic tests, concerns for genetic discrimination and insurance coverage, and the emotional burden of a cancer diagnosis. A limitation of our analysis of the uptake of cascade testing is that testing could have been conducted through other laboratories; however, this is likely small because of the financial advantage (no charge) of completing the testing through the laboratory used in this study.

Aspects of this study merit discussion. Its considerable strength includes a prospective design with a diverse population of race/ethnicity. The participation rate of African Americans/Blacks in this study contributes to the field of knowledge regarding PGV, VUS, and incremental findings compared to guidelines in this population. This study utilized pre-test genetic education via a pre-recorded video and post-test telegenetic counseling from central sites at Mayo Clinic (Arizona and Florida) in a community oncology clinic with a diverse racial/ethnic population, showing that this will be a practical approach for practices without onsite genetic counselors. Results are generalizable to other community cancer practices, given the recruitment of patients regardless of cancer type, stage, age of diagnosis, and family history of cancer. This study was uniquely able to ascertain the effectiveness of current clinical practice guidelines and the uptake of cascade FVT in a racially diverse population. Limitations include single-center recruitment, moderate sample size, and lack of long-term follow-up to assess cancer-related mortality and morbidity. Family history interpretation and the necessity for testing were determined by expert reviewers using guidelines that frequently changed during the study. The cascade FVT portion of the study relied solely on communicating test results to the family members through the proband.

## Conclusions

In conclusion, the results of this prospective cohort study in a racially diverse community population support the use of multi-gene panel testing for hereditary cancer assessment. Traditional barriers to onsite genetic counseling can be supplanted by the application of models of pre-test education using pre-recorded videos and post-test counseling through telegenic counseling from area academic medical centers. Though PGV prevalence was similar in African American/Black populations compared to others, the rate of VUS and under-performance of clinical practice guidelines raises concerns about the limitations resulting from the historical paucity of genomics data in minority populations. This study offers significant insight into the performance of MGPT in broad, racially diverse community populations. It has far-reaching implications for the clinical implementation of genomic medicine in current oncology practices.

## Appendices

**Table 6:** Potential Therapies and/or Clinical Trials Based on PGVs Detected.

Penetrance	Gene	# Patients	Precision therapy	Clinical Trial	Management guidelines	NCT
High	BRCA1	2	Y	Y	Y	NCT02286687, NCT04171700
	BRCA2	3	Y	Y	Y	NCT02286687, NCT04171700
	CDKN2A	1			Y	
	MEN1	1			Y	
	PALB2	1		Y	Y	NCT02401347, NCT04171700
	SDHA	1			Y	
	TP53	2			Y	
Moderate	ATM	1	Y	Y	Y	NCT02286687, NCT02401347
	BRIP1	1		Y	Y	NCT02401347, NCT04171700
	CDKN1B	1				
	CHEK2	3		Y	Y	NCT02401347
	HOXB13	1				
	MITF	2			Y	
	RAD51C	3		Y	Y	NCT02401347, NCT04171700
Low						
	MUTYH mono	1			Y	
Recessive						
	RECQL4	1				
